# Transcriptional Profiling of Insulin-like Growth Factor Signaling Components in Embryonic Lung Development and Idiopathic Pulmonary Fibrosis

**DOI:** 10.3390/cells11121973

**Published:** 2022-06-20

**Authors:** Vahid Kheirollahi, Ali Khadim, Georgios Kiliaris, Martina Korfei, Margarida Maria Barroso, Ioannis Alexopoulos, Ana Ivonne Vazquez-Armendariz, Malgorzata Wygrecka, Clemens Ruppert, Andreas Guenther, Werner Seeger, Susanne Herold, Elie El Agha

**Affiliations:** 1 Department of Medicine II, Internal Medicine, Pulmonary and Critical Care, Universities of Giessen and Marburg Lung Center (UGMLC), Member of the German Center for Lung Research (DZL), Justus-Liebig University Giessen, 35392 Giessen, Germany; vkheirollahi@gmail.com (V.K.); ali.khadim@innere.med.uni-giessen.de (A.K.); georgios.kiliaris@innere.med.uni-giessen.de (G.K.); martina.korfei@innere.med.uni-giessen.de (M.K.); margarida.barroso@innere.med.uni-giessen.de (M.M.B.); ioannis.alexopoulos@innere.med.uni-giessen.de (I.A.); ana.i.vazquez-armendariz@innere.med.uni-giessen.de (A.I.V.-A.); malgorzata.wygrecka@innere.med.uni-giessen.de (M.W.); clemens.ruppert@innere.med.uni-giessen.de (C.R.); andreas.guenther@innere.med.uni-giessen.de (A.G.); werner.seeger@innere.med.uni-giessen.de (W.S.); susanne.herold@innere.med.uni-giessen.de (S.H.); 2 Department of Medicine V, Internal Medicine, Infectious Diseases and Infection Control, Universities of Giessen and Marburg Lung Center (UGMLC), Member of the German Center for Lung Research (DZL), Justus-Liebig University Giessen, 35392 Giessen, Germany; 3 Cardio-Pulmonary Institute (CPI), Justus-Liebig University Giessen, 35392 Giessen, Germany; 4 Institute for Lung Health (ILH), Justus-Liebig University Giessen, 35392 Giessen, Germany

**Keywords:** IGF1, IGF1R, lung development, bleomycin-induced pulmonary fibrosis, idiopathic pulmonary fibrosis

## Abstract

Insulin-like growth factor (IGF) signaling controls the development and growth of many organs, including the lung. Loss of function of *Igf1* or its receptor *Igf1r* impairs lung development and leads to neonatal respiratory distress in mice. Although many components of the IGF signaling pathway have shown to be dysregulated in idiopathic pulmonary fibrosis (IPF), the expression pattern of such components in different cellular compartments of the developing and/or fibrotic lung has been elusive. In this study, we provide a comprehensive transcriptional profile for such signaling components during embryonic lung development in mice, bleomycin-induced pulmonary fibrosis in mice and in human IPF lung explants. During late gestation, we found that *Igf1* is upregulated in parallel to *Igf1r* downregulation in the lung mesenchyme. Lung tissues derived from bleomycin-treated mice and explanted IPF lungs revealed upregulation of IGF1 in parallel to downregulation of IGF1R, in addition to upregulation of several IGF binding proteins (IGFBPs) in lung fibrosis. Finally, treatment of IPF lung fibroblasts with recombinant IGF1 led to myogenic differentiation. Our data serve as a resource for the transcriptional profile of IGF signaling components and warrant further research on the involvement of this pathway in both lung development and pulmonary disease.

## 1. Introduction

The insulin-like growth factor (IGF) family consists of two ligands (IGF1 and IGF2), two receptors (IGF1R and IGF2R), six IGF-binding proteins (IGFBP1-6) and one IGFBP-related protein (IGFBP-rP1 or IGFBP7). IGFBPs regulate the bioavailability of IGF ligands in the blood stream. As the name suggests, IGF shares structural homology with insulin and can therefore bind to and activate the insulin receptor, albeit with lower affinity than insulin. Although it is expressed in most tissues, IGF1 is mainly produced by the liver upon growth hormone (GH) stimulation. In fact, IGF1 functions as a growth hormone and *IGF1* deficiency is linked to dwarfism in mice and humans [1,2,3,4]. IGF1 is regarded as the natural ligand for IGF1R, and therefore transduces mitogenic and survival signals by activating MAPK, PI3K/AKT and mTOR signaling pathways [5]. On the other hand, IGF2 binds to IGF1R and IGF2R, with the latter functioning as a clearance receptor for IGF2 [6].

IGF signaling has been shown to be involved in murine lung organogenesis. *Igf1*-knockout newborn pups suffer from disproportional lung hypoplasia and die due to respiratory distress [1,7]. These mutants display thickened mesenchyme, alterations in extracellular matrix (ECM) protein deposition, thin smooth muscle and dilated blood vessels, indicating delayed lung development [1,7]. Using a hypomorphic *Igf1r^neo^* allele that yields an 80% reduction in *Igf1r* expression, it was shown that reduced expression of *Igf1r* does not lead to an obvious phenotype [8]. On the other hand, *Igf1r*-knockout mouse embryos suffer from general organ hypoplasia including severe lung hypoplasia and underdeveloped diaphragms. These mutants die shortly after birth due to respiratory distress [1,8]. Histological analysis during late gestation showed that *Igf1r*-knockout mouse lungs display thickened intersaccular mesenchyme and delayed development [8]. *Igf2*-kockout pups also display delayed lung development at the end of gestation [9]. These pups display lower plasma corticosterone levels, and supplementing pregnant mice carrying *Igf2*-knockout pups with corticosterone rescues delayed lung development [9].

IGF1/IGF1R signaling has also been studied in the context of mouse models of lung injury and repair. For instance, *Igf1r* deficiency improves survival and ameliorates lung injury in response to hyperoxia [10]. IGF1R signaling controls the kinetics of cell proliferation and differentiation during airway epithelial regeneration [11]. Moreover, smooth muscle-derived IGF1 has been implicated in pulmonary hypertension development in response to hypoxia [12]. Last but not least, intervention with monoclonal antibodies against IGF1R attenuates bleomycin-induced pulmonary fibrosis [13].

Lipofibroblasts are adipocyte-like cells that not only transfer triglycerides to adjacent type 2 alveolar epithelial cells (AT2) to assist them in the process of surfactant production, but are also regarded as a niche that maintains AT2 stemness [14,15,16]. We have previously shown that lipofibroblasts are a source of myofibroblasts in lung fibrosis and that myofibroblast-to-lipofibroblast transdifferentiation represents a route for fibrosis resolution [17,18,19]. Since insulin signaling is integral to the differentiation of preadipocytes to adipocytes [20], and given that decreased IGF signaling is linked to impaired alveolar maturation, the question arose whether IGF signaling is involved in lipofibroblast formation. In this study, we provide a transcriptional profile for IGF family components at various stages of embryonic murine lung development, murine lung fibrosis and lung samples derived from idiopathic pulmonary fibrosis (IPF) patients. We also provide in vitro data on the effect of recombinant IGF1 on primary cultures of human IPF lung fibroblasts.

## 2. Materials and Methods

### 2.1. Animal Experiments

All animal experiments were approved by the local authorities. Mice were housed in a specific-pathogen-free (SPF) environment with free access to food and water. RjOrl:SWISS mice were obtained from Janvier Labs and timed pregnant females were used to collect embryonic tissues at the indicated timepoints (Approval number: 437_M). In bleomycin experiments, C57BL6/J female mice were subjected to a single intratracheal injection of saline (SAL; *n* = 6) or bleomycin (BLM; 2.5 U/Kg), and mice were euthanized after 7 (BLM d7; *n* = 5) or 14 days (BLM d14; *n* = 5) (Approval number: Gi20/10, No.: 109/2011, JLU-number: 594_GP). Protein lysates were used for western blotting and lung sections were used for histological analysis. Lung tissues were also used for RNA extraction and gene expression analysis.

### 2.2. Human-Derived Lung Material

Lung tissues and interstitial fibroblasts were isolated from explanted IPF and control lungs, collected in frame of the European IPF registry (eurIPFreg) and provided by the UGMLC Giessen Biobank, member of the DZL Platform Biobanking. The Ethics Committee of the Justus-Liebig University has approved the biospecimen collection of the UGMLC/DZL biobank under the ethics vote number 58/15. The patients have been informed and given their written consent for the use of biospecimen for research purposes. All studies and procedures to obtain human specimen were conducted according to the Declaration of Helsinki.

### 2.3. Primary Culture of Murine Lung Fibroblasts

Primary murine lung fibroblasts were cultured by differential adhesion as previously described [21]. Embryonic lungs were harvested and minced into fine pieces using blades, followed by digestion in 0.5% (*w*/*v*) collagenase IV (Thermo Fisher Scientific, Schwerte, Germany) for 45 min with slight agitation. Digested suspensions were then aspirated through 18G, 20G and 24G needles before being passed through 70 µm and 40 µm cell strainers (Sarstedt, Nümbrecht, Germany). Single-cell suspensions were allowed to adhere onto six-well plates for 17 min before washing with PBS. Cells were allowed to grow in DMEM (low glucose, GlutaMAX^TM^ Supplement, pyruvate) (Thermo Fisher Scientific) supplemented with 10% bovine calf serum (BCS) (Thermo Fisher Scientific) for the next 24 h before fresh culture medium was added.

### 2.4. Primary Culture of Human Lung Fibroblasts

Primary human lung fibroblasts derived from IPF patients were maintained in DMEM (Thermo Fisher Scientific) supplemented with 10% BCS (Thermo Fisher Scientific). Cells between passages three and five were used for experiments. Three hundred thousand cells were plated per well in six-well plates. After 24 h, cells were starved by replacing culture media by serum-free media for 24 h. Cells were then treated with recombinant human IGF1 (rhIGF1, 250 ng/mL) (R&D systems, Wiesbaden, Germany) or vehicle (phosphate-buffered saline, PBS) for 72 h. Cells derived from the same patients were used as controls. In the designated experiments, cells were cultured on CytoSoft plates with elastic modulus of 16 kPa (Sigma-Aldrich, St. Louis, MO, USA).

### 2.5. RNA Extraction, cDNA Synthesis and qPCR

RNeasy mini kit (Qiagen, Hilden, Germany) was used for RNA extraction followed by cDNA synthesis using Quantitect reverse transcription kit (Qiagen) according to the manufacturer’s protocol. Quantitative real-time PCR (qPCR) was carried out using PowerUp SYBR green master mix (Thermo Fisher Scientific) and LightCycler 480 II machine (Roche Applied Science, Mannheim, Germany). Primer sequences are listed in Table 1.

### 2.6. Histology, Immunohistochemistry and Fluorescent Staining

Formalin-fixed, paraffin-embedded mouse and human lung tissues were subjected to immunohistochemistry as we previously described [17,22,23] using antibodies against IGF1 (Abcam, Berlin, Germany) (1:500), IGF1R (Sigma-Aldrich; 1:75) and TTF1 (Abcam; 1:100) and ZytoChem Plus AP Kit (Zytomed Systems, Berlin, Germany). Hematoxylin/eosin and Masson Goldner stains were carried out according to standard procedures. LipidTOX staining was carried out as previously described [18] and imaged using EVOS Cell Imaging System (Thermo Fisher Scientific).

### 2.7. Western Blotting

Western blots were carried out according to standard procedures. Antibodies against PAI-1 (R&D Systems) and mature COL1A1 (Meridian Life Science, Luckenwalde, Germany) were used.

### 2.8. Figure Assembly and Statistical Analysis

Quantitative data were assembled and analyzed using GraphPad Prism 9 (GraphPad Software, San Diego, CA, USA). The normality test was carried out whenever the number of biological samples allowed. Outliers in the data from human-derived lung material were detected using the ROUT method. For comparing two groups, t-test or Mann–Whitney test was carried out. One-way ANOVA was used to compare more than two groups. The number of biological samples (depicted as *n*) is shown in the corresponding figure legends. Figures were assembled using Adobe Illustrator (Adobe, San Jose, CA, USA).

## 3. Results

### 3.1. Transcriptional Profile of IGF Signaling Components during Embryonic Lung Development

**Figure 1:**
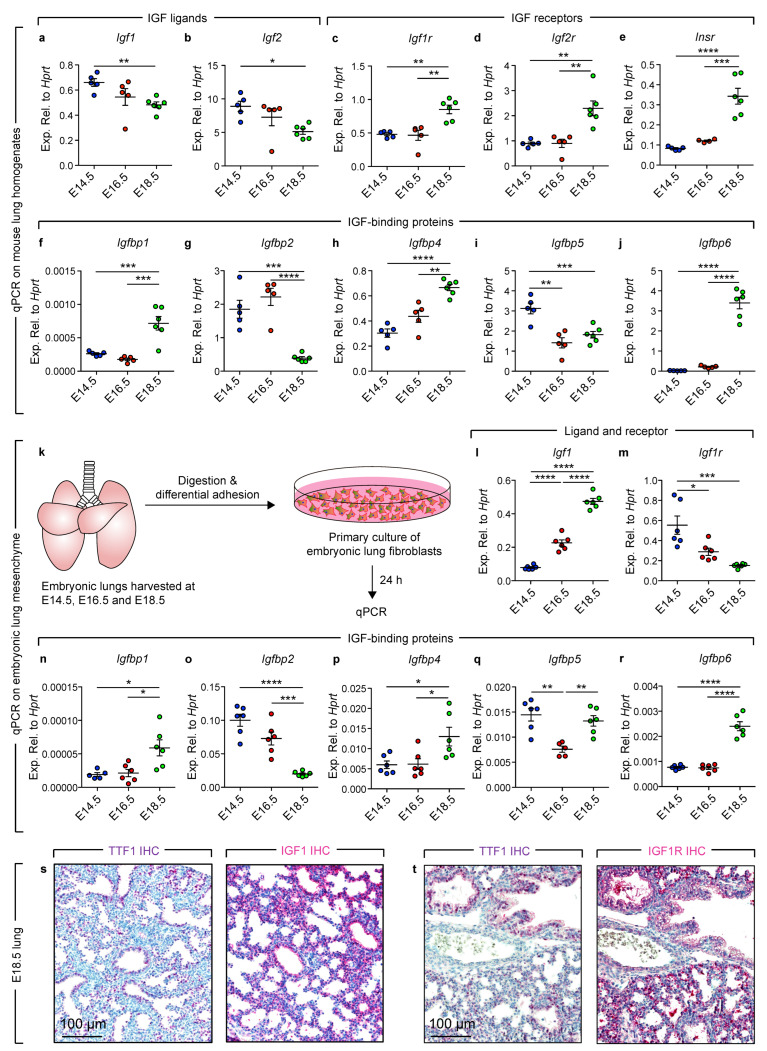
Expression profile of IGF signaling components during lung development. (**a**–**j**) qPCR on lung homogenates at the indicated developmental stages; (**k**) Scheme for experimental design; (**l**–**r**) qPCR on primary mesenchymal cells at the indicated developmental stages. (**s**) Immunohistochemistry for TTF1 and IGF1 on E18.5 mouse lungs. (**t**) Immunohistochemistry for TTF1 and IGF1R on E18.5 mouse lungs. One-way ANOVA was used to compare the means. (**a**–**j**) E14.5: *n* = 5, E16.5: *n* = 5, E18.5: *n* = 6; (**l**–**r**) E14.5: *n* = 5–6, E16.5: *n* = 5–6, E18.5: *n* = 6. * *p* < 0.05, ** *p* < 0.01, *** *p* < 0.001, **** *p* < 0.0001.

To explore the expression pattern of IGF signaling components during embryonic lung development, embryos were collected from timed pregnant mice and RNA was isolated from lung homogenates and subjected to qPCR at multiple developmental stages (E14.5: Pseudoglandular stage; E16.5: End of pseudoglandular stage-beginning of canalicular stage; E18.5: Saccular stage) (Figure 1). These timepoints were chosen because various epithelial and mesenchymal cell lineages such as alveolar epithelial cells and lipofibroblasts start to emerge around E16.5 [21,24,25,26]. The results revealed that *Igf1* and *Igf2*, encoding the main IGF ligands, showed a decline from E14.5 to E18.5 (Figure 1a,b). On the other hand, the expression levels of IGF receptors, *Igf1r* and *Igf2r*, showed a 1.77- and a 2.57-fold increase, respectively (Figure 1c,d). *Insr*, encoding the insulin receptor gene, showed a similar upregulation at E18.5 (Figure 1e). Analysis of the expression levels of *Igfbp* genes showed a mixed pattern (Figure 1f–j). *Igfbp1*/*4*/*6* showed significant upregulation at E18.5 compared with E14.5 and E16.5. Conversely, *Igfbp2* showed significant downregulation at E18.5, while *Igfbp5* showed significant downregulation at E16.5 and E18.5 compared with E14.5.

The data described in Figure 1a–j reflect gene expression in whole-lung homogenates containing a mixture of endoderm- and mesoderm-derived cells such as epithelial, endothelial and mesenchymal cells. In order to investigate the expression pattern of these genes exclusively in mesenchymal cells, cell suspensions were prepared and subjected to differential adhesion as previously described [21]. Mesenchymal cells were allowed to grow for 24 h (Figure 1k). While *Igf1* showed gradual upregulation from E14.5 to E18.5 (around 6-fold increase at E18.5 compared with E14.5) (Figure 1l), *Igf1r* showed an opposite pattern where its expression levels decreased by 1.9 folds at E16.5 and 3.7 folds at E18.5 (Figure 1m). The expression pattern of *Igfbp* genes was very similar in cultured mesenchymal cells and lung homogenates (Figure 1n–r vs. Figure 1f–j). Immunohistochemistry on E18.5 lung sections showed that IGF1 immunoreactivity could be detected in both the epithelium and the mesenchyme, although the signal was stronger in the epithelium (Figure 1s). In agreement with the qPCR data, IGF1R was detected in epithelial cells rather than mesenchymal cells at this stage (Figure 1t). Collectively, these data indicate that *Igf1* is upregulated while *Igf1r* is downregulated in the lung mesenchyme during last gestation. Moreover, *Igfbp1/4/6* are upregulated while *Igfbp2* is downregulated in this lung compartment.

### 3.2. Increased Igf1 Expression during Fibrosis Development in Bleomycin-Induced Lung Injury

**Figure 2:**
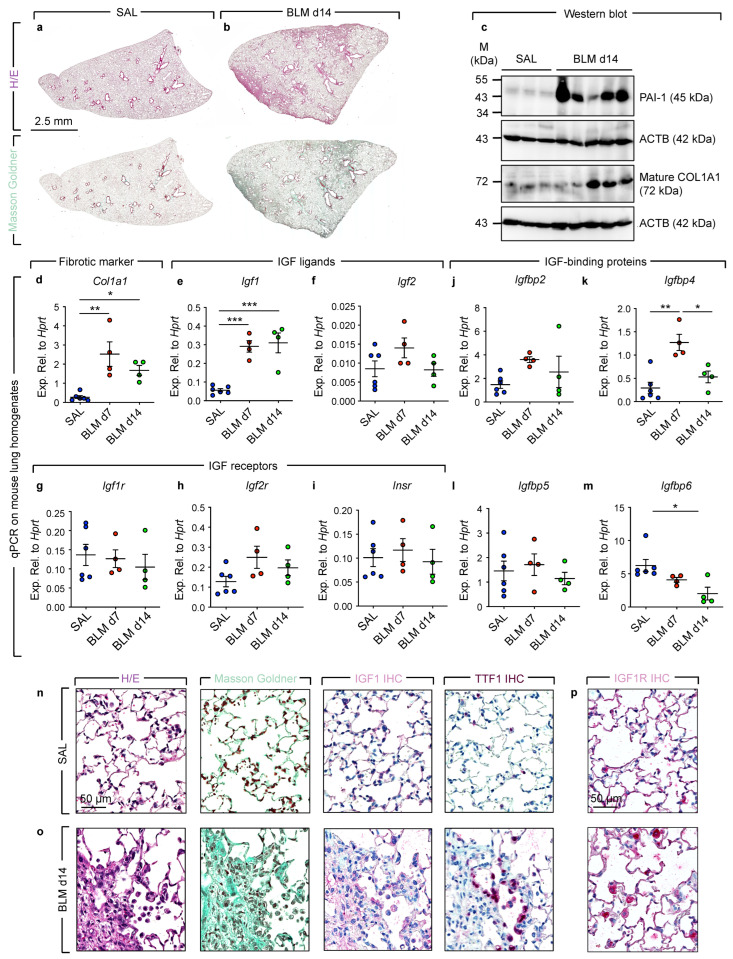
Alteration of IGF signaling in the bleomycin model of lung fibrosis. (**a**,**b**) Hematoxylin/eosin and Masson Goldner staining showing clear fibrosis in bleomycin-treated mouse lungs compared with saline-treated controls. (**c**) Western blot for PAI-1, COL1A1 and ACTB. (**d**–**m**) qPCR on lung homogenates at the indicated timepoints. (**n**,**o**) Hematoxylin/eosin stain, Masson Goldner stain, IGF1 immunohistochemistry and TTF1 immunohistochemistry on saline- and bleomycin-treated mouse lungs. (**p**) IGF1R immunohistochemistry on saline- and bleomycin-treated mouse lungs. One-way ANOVA was used to compare the means. SAL: *n* = 6, BLM d7: *n* = 4, BLM d14: *n* = 4. * *p* < 0.05, ** *p* < 0.01, *** *p* < 0.001. SAL: Saline; BLM: Bleomycin; IHC: Immunohistochemistry.

The expression levels of IGF signaling components were also examined in lung homogenates from bleomycin-treated mice at day 7 (end of acute lung injury/inflammatory phase—beginning of fibrotic phase) and day 14 (peak of fibrosis) and were compared with saline controls. Firstly, histological analysis using hematoxylin/eosin and Masson Goldner stains confirmed the presence of fibrosis at day 14 (Figure 2a,b). At the transcriptional level, *Col1a1* showed significant upregulation at days 7 and 14 (Figure 2d). Such upregulation was also confirmed by western blotting (Figure 2c). *Igf1* appeared to mimic *Col1a1* expression pattern where it showed significant upregulation, while *Igf2* did not show significant alterations (Figure 2e,f).

Analysis of genes encoding IGF receptors did not show significant changes in bleomycin-treated lungs (Figure 2g–i). During the course of injury, *Igfbp4* showed transient upregulation at day 7 before normalizing at day 14 (Figure 2k), while *Igfbp6* showed significant downregulation at day 14 (Figure 2m). On the other hand, *Igfbp2/5* did not show significant changes (Figure 2j,l). The upregulation of *Igf1* at the transcriptional level was also reflected at the protein level by immunohistochemistry, where IGF1 immunoreactivity was observed in areas of collagen deposition as well as in epithelial cells (Figure 2n,o). Finally, immunohistochemistry for IGF1R showed a strong signal in epithelial cells (AT2 and bronchial epithelium) in both saline and bleomycin-treated lungs (Figure 2p).

### 3.3. IGF1 Expression Is Elevated in IPF Lungs

**Figure 3:**
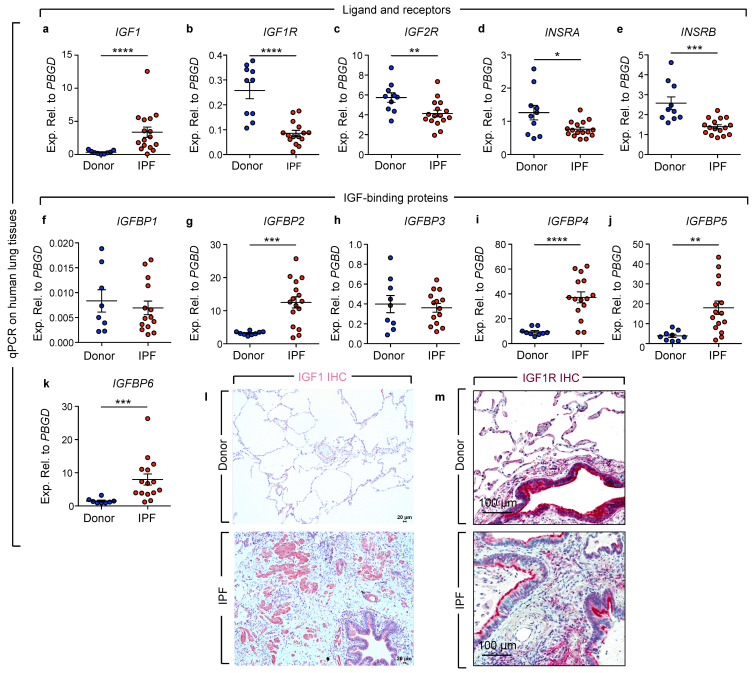
Alteration of IGF1 signaling in idiopathic pulmonary fibrosis. (**a**–**k**) qPCR on homogenates of lung explants derived from donor or IPF patients; (**l**,**m**) Immunohistochemistry for IGF1 and IGF1R on donor and IPF lung sections. *t*-test (**b**–**e**,**g**–**j**) or Mann–Whitney test (**a**,**f**,**k**) was performed to compare the groups. Donor: *n* = 8–10, IPF: *n* = 14–16. * *p* < 0.05, ** *p* < 0.01, *** *p* < 0.001, **** *p* < 0.0001. IHC: Immunohistochemistry.

Lung homogenates from donors and IPF patients were subjected to gene expression analysis. *IGF1* showed a strong 9.9-fold upregulation (Figure 3a) while *IGF1R* showed a 3-fold downregulation in IPF samples compared with donors (Figure 3b).

*INSRA/B* showed significant downregulation in IPF samples compared with donors (Figure 3d,e). While *IGF2* transcripts could not be detected in human lung tissues, *IGF2R* showed significant downregulation in IPF lungs compared with donor lungs (Figure 3c). No significant changes were observed in the expression levels of *IGFBP1/3* while *IGFBP2/4/5/6* showed significant upregulation in IPF lungs compared with donor lungs (3.9, 4.8, 5.6 and 6.4 folds, respectively) (Figure 3f–k). Finally, immunohistochemistry showed significant upregulation of IGF1 in IPF lung explants compared with donors, where IGF1 immunoreactivity was robust in alveolar and bronchiolar epithelial cells as well as in areas of dense fibrosis (Figure 3l). In contrast, IGF1 immunoreactivity was sparse in any cells of normal donor lungs (Figure 3l). Immunohistochemistry for IGF1R, on the other hand, showed a strong signal in bronchiolar cells in donor lungs, and the signal was weaker in IPF lungs (Figure 3m).

### 3.4. Treatment of Human Lung Fibroblasts with Recombinant IGF1 Leads to Loss of Lipid Droplets

**Figure 4:**
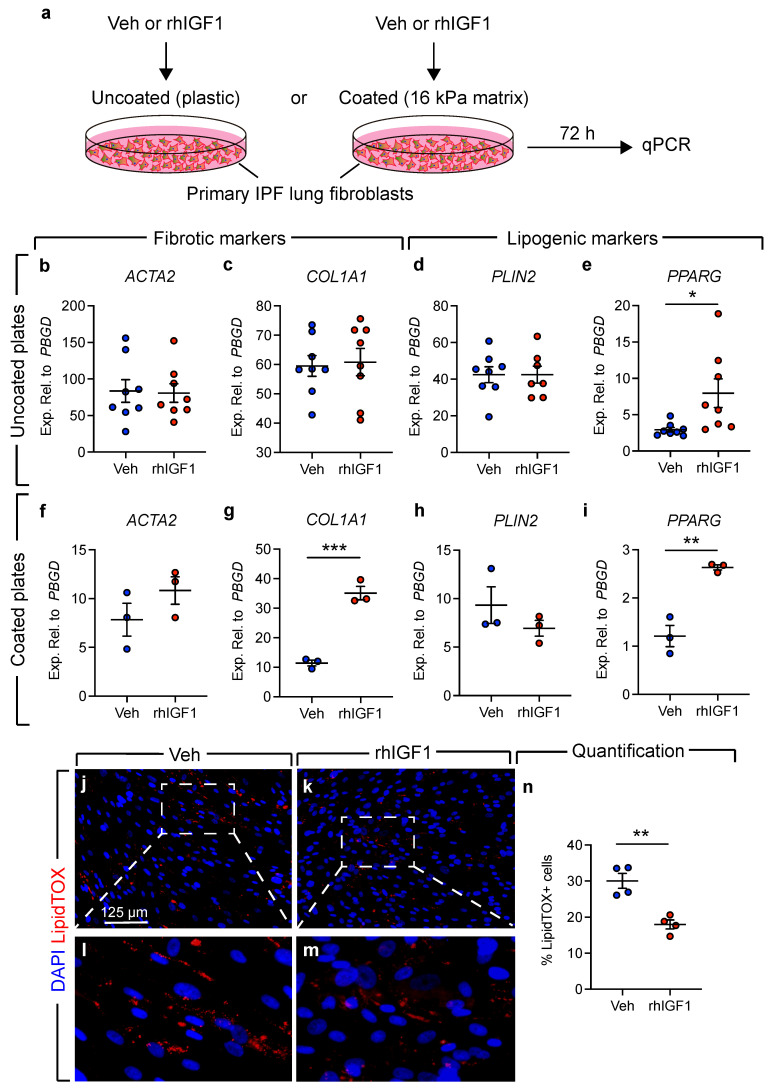
Effect of recombinant IGF1 treatment on primary human lung fibroblasts. (**a**) Scheme for experimental design. (**b**–**e**) qPCR on primary IPF lung fibroblasts cultured on uncoated plates and treated with vehicle or recombinant human IGF1. (**f**–**i**) Similar analysis using coated plates. (**j**–**m**) Neutral lipid stain on primary IPF lung fibroblasts cultured on uncoated plates. Nuclei are stained with DAPI. (**n**) Quantification of LipidTOX staining. rhIGF1: Recombinant human IGF1; Veh: Vehicle. t-test was used to compare the means. (**b**–**e**) Veh: *n* = 8, rhIGF1: *n* = 7–8; (**f**–**i**) *n* = 3 per group; (**n**) *n* = 4 per group. * *p* < 0.05, ** *p* < 0.01, *** *p* < 0.001.

Due to the link between IGF1 signaling and lung fibrosis, we decided to investigate whether treatment with recombinant human IGF1 (rhIGF1) affects the expression levels of lipofibroblast and myofibroblast markers using primary cultures of human IPF lung fibroblasts (Figure 4). Given the impact of matrix stiffness on myofibroblast differentiation [27,28,29], we opted to use two culture conditions: Cells cultured on uncoated plates (plastic) and cells cultured on a 16 kPa matrix that mimics the pathophysiological setting linked to the induction of alpha smooth muscle actin (ACTA2) expression in fibrotic tissue (estimated around 20 kPa) [28,30] (Figure 4a). Treatment of cells cultured on uncoated plates with rhIGF1 did not lead to significant alteration in the expression levels of *ACTA2*, *COL1A1* or *PLIN2* (Figure 4b–d). However, it led to significant upregulation of *PPARG* (Figure 4e). On the other hand, treatment of cells cultured on coated plates with rhIGF1 led to upregulation of the myofibroblast markers *ACTA2* (trend) and *COL1A1* (significant) in parallel to significant upregulation of *PPARG* (Figure 4f,g,i). The expression levels of *PLIN2* were not significantly changed in response to rhIGF1 treatment (Figure 4h). To confirm whether the lipogenic properties were significantly influenced by rhIGF1 treatment, cells cultured on uncoated plates were stained with the neutral lipid dye, LipidTOX (Figure 4j–m). Quantification did not show induction of adipogenesis but rather showed significant loss of lipid droplets in response to rhIGF1 treatment (Figure 4n).

## 4. Discussion

IGF signaling is involved in many developmental and pathological processes, but its regulation remains poorly understood. IGF1/ IGF2, their receptors, insulin signaling components and several IGFBPs contribute to the complexity of this signaling pathway. In this work, we report the transcriptional profile of IGF signaling components during embryonic murine lung development, murine lung fibrosis and human IPF.

A link between IGF signaling and alveolar fibroblast subsets has already been discussed. For instance, a previous study suggested that *Igf1r* is downregulated in lipofibroblasts rather than non-lipofibroblasts after alveolarization, an event that correlates with apoptosis of these cells, thus hinting to a possible role for IGF1R signaling in lipofibroblast survival [31]. Our gene expression analysis showed that *Igf1* is significantly upregulated in mesenchymal cells while *Igf1r* is significantly downregulated during late gestation. Based on available single-cell RNA-seq data [32], myofibroblasts and smooth muscle cells are the main source of *Igf1* at E16.5 while *Igf1r* is more ubiquitously expressed. The significant increase in *Igf1* expression at E18.5 might be due to the increasing number of myofibroblast progenitors that are required for postnatal septation [33]. Interestingly, a previous study showed that in newborn lungs, platelet-derived growth factor receptor alpha-positive (PDGFRα+) alveolar fibroblasts (typically refer to myofibroblasts at this developmental stage) secrete IGF1, which acts on innate lymphoid cell (ILC) progenitors to promote their proliferation [34].

Interestingly, we could detect *Igf1* and *Igf1r*, but not *Igf2* or *Igf2r,* transcripts in our primary cultures of murine lung fibroblasts at various developmental stages. So far, the literature has mainly focused on the role of the IGF1/IGF1R axis in disease and repair. Nevertheless, recent work suggests that like IGF1, IGF2 exerts profibrotic effects and promotes myofibroblast differentiation [35]. It is important to mention that IGF1/2 are often bound to IGFBPs, which regulate the bioavailability of these ligands and suppress or promote IGF signaling in a tissue- and cell-context-dependent manner [36]. The similar expression patterns of *Igfbp1, Igfbp4* and *Igfbp6* between E14.5 and E18.5 suggest that they might play similar roles during this period of lung development. On the other hand, it was reported that increased levels of *Igfbp2* result in proliferation arrest of epithelial cells in the lung [37]. This might explain the reduced levels of *Igfbp2* in lung homogenates at late stages (between E16.5 and E18.5), as this coincides with the expansion of alveolar epithelial progenitors.

IGF signaling is involved in controlling glucose and lipid metabolism and is therefore altered in diabetes mellitus. Serum levels of IGF1 were reported to be elevated in type II diabetes mellitus (T2DM) [38]. Whether such dysregulation in the abundance of IGF1 is a cause or consequence of diabetes remains unclear. Metabolic alterations have also been reported in patients suffering from IPF [39,40], and T2DM might be risk factor for IPF [41,42]. Among the metabolic pathways affected in IPF lungs are sphingolipids, arginine, energy (including glucose, fatty acid and citric acid metabolism), bile acid, heme and glutamate/aspartate [39]. Some of these pathways, particularly those related to glucose and fatty acid metabolism can be regulated by IGF1 signaling and might therefore be relevant for IGF1 research in the context of IPF.

We and others have already shown a potent antifibrotic effect for the antidiabetic compound metformin in the lung [18,43,44,45]. We have shown that metformin, as well as the PPARγ agonist rosiglitazone, accelerate the resolution of pulmonary fibrosis at least partly via inducing the transdifferentiation of collagen-secreting myofibroblasts into pro-alveologenic lipofibroblasts [17,18]. Given the parallels between lipofibroblasts and mature adipocytes, and since insulin signaling is critical for adipogenic differentiation, we tested the possibility that IGF1 plays a similar effect in promoting lipofibroblast formation. Our data, however, do not support this scenario. On the contrary, treatment of primary human IPF lung fibroblasts exacerbated the myofibroblast phenotype and led to the loss of lipid droplets in these cells. These data using human-derived lung samples agree with the gene expression analysis carried out of lung homogenates from bleomycin-challenged mice, where *Igf1* showed a similar expression pattern as *Col1a1* during fibrosis formation. Our data are therefore in line with previous reports showing that blocking IGF1R signaling ameliorates bleomycin-induced pulmonary fibrosis in experimental mice [13]. Our gene expression profiling of IGFBPs in IPF and donor lung explants showed that *IGFBP2,4,5,6* are upregulated while *IGFBP1,3* are unaltered. Some of these IGFBPs have previously been shown to be upregulated in IPF and contribute to ECM protein deposition [46,47,48]. IGFBP2, in particular, has been proposed as a biomarker for IPF [48].

One last aspect is the effect of matrix stiffness on the phenotype of fibroblasts and whether it modulates the response of these cells to rhIGF1 treatment, particularly in terms of myogenic versus lipogenic differentiation. It has been shown that mouse lung fibroblasts grown on soft substrate (<0.1 kPa), but not stiff substrate, upregulate *Acta2* when treated with recombinant IGF1. The induction of *Col1a1* expression was reported for both conditions (using mouse lung fibroblasts) [28]. Here, we cultured primary human IPF lung fibroblasts on either uncoated plates or 16 kPa substrate. While rhIGF1 showed a clear profibrotic effect on cells cultured on 16 kPa matrix, the corresponding transcriptional changes were not evident in cells grown on uncoated plates. This indicates that a (patho)physiological matrix might be important to “capture” the transcriptomic changes occurring during active myogenic differentiation. One surprising finding was the significant upregulation of *PPARG*, the master regulator of adipogenesis, under both experimental setups. Nevertheless, we could detect significant loss of lipid droplets in cells treated with rhIGF1, which fits with the model of lipogenic-to-myogenic differentiation. Whether IGF1 signaling has a direct impact on lipofibroblasts in vivo warrants further investigation.

In summary, we observed distinct expression patterns for IGF signaling components in different compartments of the lung during murine embryonic development. In the murine model of bleomycin-induced pulmonary fibrosis, we observed upregulation of *Igf1*, transient upregulation of *Igfbp4* and downregulation of *Igfbp6*. The genes encoding IGF1 and several IGFBPs are upregulated in IPF while those encoding IGF and insulin receptors are downregulated. Finally, our data using primary cultures of IPF lung fibroblasts confirm the profibrotic effect of IGF1, and hint to a possible impact on lipofibroblasts in lung fibrosis.

## Figures and Tables

**Table 1:** Primers used for qPCR.

Primer Name	Sequence (5′-3′)
hACTA2 Fwd	CTGTTCCAGCCATCCTTCAT
hACTA2 Rev	TCATGATGCTGTTGTAGGTGGT
hCOL1A1 Fwd	ATGTTCAGCTTTGTGGACCTC
hCOL1A1 Rev	CTGTACGCAGGTGATTGGTG
hIGF1 Fwd	TGTGGAGACAGGGGCTTTTA
hIGF1 Rev	ATCCACGATGCCTGTCTGA
hIGF1R Fwd	GAGAATTTCCTTCACAATTCCATC
hIGF1R Rev	CACTTGCATGACGTCTCTCC
hIGF2 Fwd	CAAACCGAGCTGGGCG
hIGF2 Rev	CACAGAGAAGCGGAGGGA
hIGF2R Fwd	TCTCCAGTGGACTGCCAAGT
hIGF2R Rev	GTGCTTAGGCCAGTCAGGTC
hIGFBP1 Fwd	AATGGATTTTATCACAGCAGACAG
hIGFBP1 Rev	GGTAGACGCACCAGCAGAGT
hIGFBP2 Fwd	AAGGGTGGCAAGCATCAC
hIGFBP2 Rev	CTGGTCCAGTTCCTGTTGG
hIGFBP3 Fwd	AACGCTAGTGCCGTCAGC
hIGFBP3 Rev	CGGTCTTCCTCCGACTCAC
hIGFBP4 Fwd	CCTCTACATCATCCCCATCC
hIGFBP4 Rev	GGTCCACACACCAGCACTT
hIGFBP5 Fwd	AGAGCTACCGCGAGCAAGT
hIGFBP5 Rev	GTAGGTCTCCTCGGCCATCT
hIGFBP6 Fwd	TGACCATCGAGGCTTCTACC
hIGFBP6 Rev	CATCCGATCCACACACCA
hINSRA Fwd	TTTTCGTCCCCAGGCCATC
hINSRB Fwd	CCCCAGAAAAACCTCTTCAGG
hINSR Rev	GTCACATTCCCAACATCGCC
hPBGD Fwd	TGTCTGGTAACGGCAATGCG
hPBGD Rev	CCCACGCGAATCACTCTCAT
hPLIN2 Fwd	TCAGCTCCATTCTACTGTTCACC
hPLIN2 Rev	CCTGAATTTTCTGATTGGCAC
hPPARG Fwd	TTGCTGTCATTATTCTCAGTGGA
hPPARG Rev	GAGGACTCAGGGTGGTTCAG
mCol1a1 Fwd	CCAAGAAGACATCCCTGAAGTCA
mCol1a1 Rev	TGCACGTCATCGCACACA
mHprt Fwd	CCTAAGATGAGCGCAAGTTGAA
mHprt Rev	CCACAGGACTAGAACACCTGCTAA
mIgf1 Fwd	AGCAGCCTTCCAACTCAATTAT
mIgf1 Rev	GAAGACGACATGATGTGTATCTTTATC
mIgf1r Fwd	AGAATTTCCTTCACAATTCCATC
mIgf1r Rev	CACTTGCATGACGTCTCTCC
mIgf2 Fwd	CGCTTCAGTTTGTCTGTTCG
mIgf2 Rev	GCAGCACTCTTCCACGATG
mIgf2r Fwd	CCTTCTCTAGTGGATTGTCAAGTG
migf2r Rev	AGGGCGCTCAAGTCATACTC
mIgfbp1 Fwd	TGGTCAGGGAGCCTGTGTA
mIgfbp1 Rev	ACAGCAGCCTTTGCCTCTT
mIgfbp2 Fwd	GCGGGTACCTGTGAAAAGAG
mIgfbp2 Rev	CCTCAGAGTGGTCGTCATCA
mIgfbp3 Fwd	GACGACGTACATTGCCTCAG
mIgfbp3 Rev	GACGACGTACATTGCCTCAG
mIgfbp4 Fwd	GACACCTCGGGAGGAACC
mIgfbp4 Rev	AAGAGGTCTTCGTGGGTACG
mIgfbp5 Fwd	GGCGAGCAAACCAAGATAGA
mIgfbp5 Rev	AGGTCTCTTCAGCCATCTCG
mIgfbp6 Fwd	GGGCTCTATGTGCCAAACTG
mIgfbp6 Rev	CCTGCGAGGAACGACACT
mInsr Fwd	TCTTTCTTCAGGAAGCTACATCTG
mInsr Rev	TGTCCAAGGCATAAAAAGAATAGTT

h: human; m: mouse.

## Data Availability

There are no deposited or supplementary data associated with this work. The data presented in this study are available in the article.
